# Life-Threatening Hemoptysis Leading to Suffocation

**DOI:** 10.7759/cureus.55001

**Published:** 2024-02-26

**Authors:** Rade Milic, Biljana Lazovic Popovic, Nensi Lalic, Sanja Sarac

**Affiliations:** 1 Pulmonology Clinic, Medical Faculty, Military Medical Academy, University of Defence, Belgrade, SRB; 2 Pulmonology Department, University Clinical Hospital Center Zemun, Belgrade, SRB; 3 Pulmonary Diseases Department, Institute for Pulmonary Diseases of Vojvodina, Sremska Kamenica, SRB; 4 Faculty of Medicine, University of Novi Sad, Novi Sad, SRB

**Keywords:** life-threatening bleeding, bronchoscopy, suffocation, bronchiectasis, hemoptysis

## Abstract

Hemoptysis represents a symptom or sign that typically causes anxiety for patients and draws the attention of the physician because it can be caused by various conditions, ranging from benign to malignant. Depending on the amount of coughed-up blood, hemoptysis can be a life-threatening condition. We present the case of a female patient with a life-threatening hemoptysis that was caused by underlying bronchiectasis and antiplatelet and anticoagulant treatments. A large blood clot was extracted from the patient’s airways using a rigid bronchoscope. Hemoptysis is a significant symptom that should be taken seriously, regardless of its size. Massive hemoptysis is an emergency condition in respiratory medicine, which requires immediate management in adequately equipped centers.

## Introduction

Hemoptysis is characterized by the coughing up of blood originating from either the tracheobronchial tree or the lung parenchyma [1]. Although the quantity of blood is limited in most patients, instances of massive hemoptysis can pose a life-threatening risk because of potential asphyxiation or excessive bleeding. Approximately 5-14% of individuals experiencing hemoptysis are anticipated to have life-threatening bleeding. Massive hemoptysis, also known as life-threatening hemoptysis, is defined in several ways. Criteria such as the presence of abnormal gas exchange or hemodynamic instability, the hourly volume of bleeding, or the total bleeding volume within 24 hours have been used for variable definitions. For example, cutoff values ranging from 100 to 1,000 mL have been proposed to determine the quantity of expelled blood in hemoptysis cases, and a consensus on any specific value has not been reached [2]. Given that the airway has a capacity for 100-200 mL of fluid, hemoptysis is considered massive if the blood loss is greater than 200 mL [3]. Mortality rates can reach 80% if hemoptysis is not appropriately managed [4,5].

We present the case of a female patient with life-threatening hemoptysis caused by underlying bronchiectasis and antiplatelet and anticoagulant treatments.

## Case presentation

A 71-year-old woman was admitted to our institution in May 2014 because she expectorated about 200 mL of blood over six hours. One month prior, she was treated in another hospital for congestive heart failure. During the hospitalization, she developed acute arterial thromboembolism in her left foot, and a surgical embolectomy was done. In March 2013, she was hospitalized for acute myocardial infarction, and percutaneous coronary intervention with stent placement was performed. At that time, mitral valve insufficiency grade 3 and atrial fibrillation were revealed, and valve replacement was recommended. The patient was treated with antiplatelet and anticoagulant drugs (acetylsalicylic acid 100 mg daily and warfarin 5 mg daily), a diuretic (hydrochlorothiazide), an angiotensin-converting enzyme inhibitor (fosinopril), a cardio-selective beta-blocker (bisoprolol), isosorbide mononitrate, and lovastatin. Physical examination on admission revealed pale skin, absolute arrhythmia, and a systolic murmur of mitral regurgitation. Other physical findings and vital parameters were normal. Initial laboratory tests revealed mild anemia. The international normalized ratio was in the therapeutic range. Hematology and chemistry analyses did not reveal clinically significant changes (Table 1).

**Table 1:** Laboratory test results

Analysis	Result	Normal value
Erythrocyte sedimentation rate (at one hour)	86 mm/h	0–15 mm/1h
Hemoglobin	103 g/L	115–165 g/L
Hematocrit	0.31 L/L	0.37–0.47 L/L
Red blood cell count	3.6x10^12^/L	3.8–5.8x10^12^/L
White blood cell count	6.9x10^9^/L	4–11x10^9^/L
Neutrophil count	4.2 x10^9^/L	1.9–8x10^9^/L
Basophil count	0.1x10^9^/L	0.0–0.2x10^9^/L
Eosinophil count	0.6 x10^9^/L	0.0–0.8x10^9^/L
Monocyte count	0.8 x10^9^/L	0.16–1.2 x10^9^/L
Lymphocyte count	1.2 x10^9^/L	0.9–5.2 x10^9^/L
Platelet count	303 x10^9^/L	160–370 x10^9^/L
Sodium	142 mmol/L	136–145 mmol/L
Potassium	4.8 mmol/L	3.5–5.1 mmol/L
Calcium	2.28 mmol/L	2.15–2-6 mmol/L
Chloride	105 mmol/L	98–111 mmol/L
Creatinine	82 µmol/L	62–115 µmol/L
Urea	6.7 mmol/L	2.5–7.5 mmol/L
Albumin	42 g/L	32–50 g/L
Aspartate transaminase	20 U/L	0–37 U/L
Alanine transaminase	25 U/L	10–49 U/L
Gamma-glutamyl transferase	28 U/L	0–73 U/L
International normalized ratio	2.18	2–4
C-reactive protein	3.9 mg/L	0–4 mg/L

**Figure 1:**
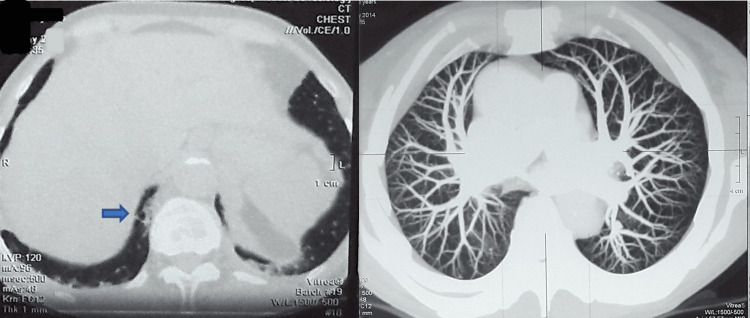
Chest multidetector computed tomography: bronchiectasis in the lower lobes (blue arrow). Bronchiectasis is most commonly a consequence of lower respiratory tract infections and can pose a risk factor for the occurrence of hemoptysis.

A multidetector computed tomography (MDCT) pulmonary angiogram revealed mild bronchiectasis in the lower lobes, but there was no sign of infiltration of the lungs, pulmonary embolism, or pathologic vascularization (Figure 1). Chest MDCT (arterial phase) was done in addition and did not detect extravasation of contrast.

**Figure 2:**
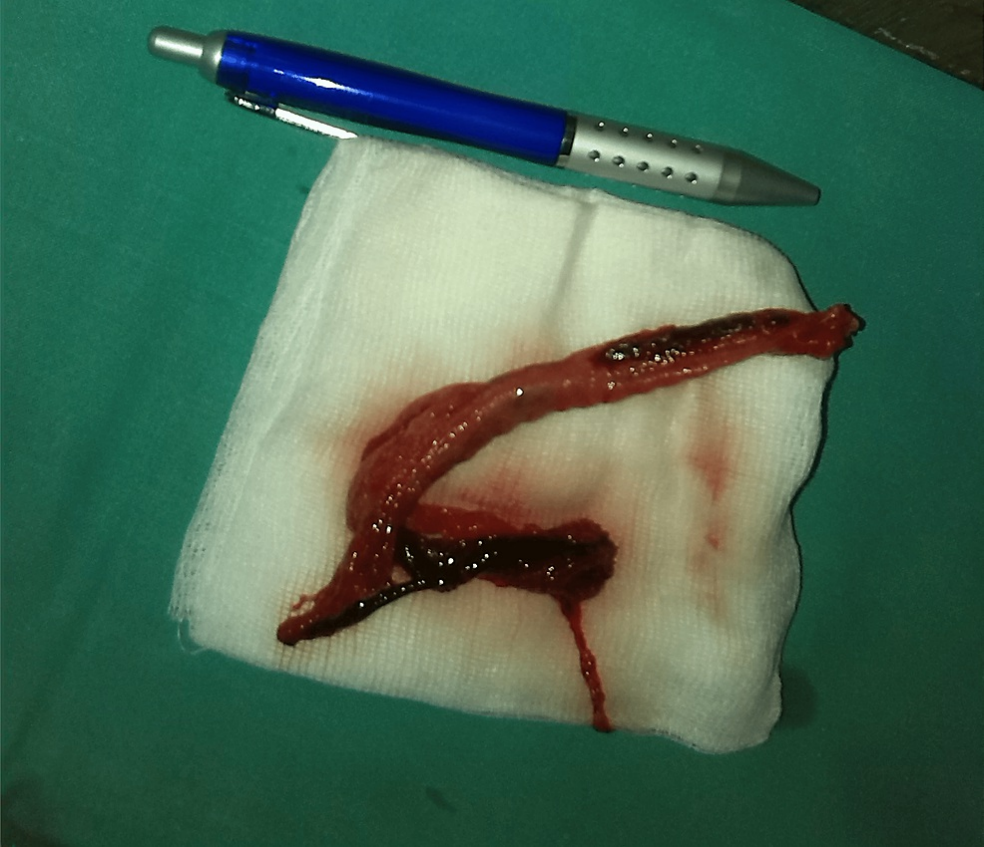
Extracted, airway-shaped blood clot

Antiplatelet and oral anticoagulant drugs were withdrawn, and no visible hemoptysis was observed during the night. The next morning, the patient became dyspneic with central cyanosis. The patient’s use of accessory respiratory muscles was visible. An emerging bronchoscopy was performed, and a giant blood clot was seen in the trachea and left principal bronchus. Extraction with a flexible video-bronchoscope was unsuccessful. The patient’s condition deteriorated, and she became soporous. Her atrial fibrillation worsened, with a ventricle rate of 180 beats per minute. Blood gas analyses revealed a global respiratory failure. The patient underwent analgosedation, a rigid bronchoscopy was performed, and the blood clot was extracted. The clot was shaped like a tracheobronchial tree (Figure 2).

After the procedure, the patient woke, and physical and neurological findings were normalized. She stayed in the hospital for the next seven days and was treated with an antibiotic (ceftriaxone 2 g daily) and low-molecular-weight heparin (nadroparin calcium 0.3 mL and 2,850 anti-Xa IU daily) without hemoptysis. She was discharged in good condition with the recommendation to continue taking a low dose of low-molecular-weight heparin (nadroparin calcium 0.3 mL and 2,850 anti-Xa IU (international units) daily) and acetylsalicylic acid 50 mg daily. The patient returned for a regular checkup after one month, and she has not had hemoptysis anymore. A control bronchoscopy revealed her tracheobronchial tree without any abnormalities.

## Discussion

Hemoptysis can be caused by various conditions. Minimal amounts of expectorated blood may be a sign of a severe disease, such as lung cancer. In contrast, massive hemoptysis may result from a benign disease, such as tuberculosis, bronchiectasis, or arterio-venous malformation. Massive hemoptysis still requires an urgent diagnostic approach, and treatment because it can be fatal. In our patient, hemoptysis was most likely caused by underlying bronchiectasis, provoked by antiplatelet and anticoagulant treatments prescribed owing to comorbidities. The hemoptysis was life-threatening as it mechanically obstructed the airways because of the patient’s large blood clot and acute asphyxia. Algorithms for massive and nonmassive hemoptysis are recommended in the literature [6-8]. In simple cases, symptomatic treatment is often sufficient, but interventional bronchoscopic procedures may be necessary (e.g., cold saline, vasoconstrictive agents, bronchoscopy-guided topical hemostatic, endobronchial biocompatible glue, endobronchial stents, endobronchial embolization using silicone spigots, laser photocoagulation, argon plasma coagulation, endobronchial valves, selective endobronchial intubation, and double-lumen tube) [5]. When and on whom bronchoscopy should be done after an initial episode of hemoptysis are important questions discussed in the CHEST editorial “Point and Counterpoint” [9]. The conclusion was that bronchoscopy is complementary to a chest CT and should be considered for all patients with massive hemoptysis and those with nonmassive hemoptysis and abnormal imaging studies or risk factors for lung cancer. Additionally, it is important to distinguish hemoptysis from pseudo-hemoptysis, such as hematemesis and bleeding from the upper airways [10]. Bronchial artery embolization may be a good method for controlling hemoptysis; however, a low clinical success rate was noted in patients with unstable hemodynamics and coagulopathy, and multiple vessel embolization was associated with a higher rate of clinical success [11]. For patients with benign lung diseases, surgical treatment is the recommended approach, with acceptable rates of morbidity and mortality. Considering elective sublobar resection, if appropriate, using video-assisted thoracoscopic surgery was advised to improve outcomes after the surgery [12]. Surgery as a life-saving procedure to resolve massive hemoptysis should be avoided because of its high mortality rate. Instead, planned, delayed surgery should be performed when possible [13].

## Conclusions

Hemoptysis, regardless of size, is a significant symptom that should be addressed. A careful diagnostic and therapeutic approach is necessary. Massive hemoptysis can be a life-threatening condition and should be managed in an adequately equipped hospital by healthcare providers who have extensive experience in treating hemoptysis. This case emphasizes the significance of early intervention and underscores the crucial role seasoned professionals play in navigating the complexities of this challenging clinical scenario.
